# Effect of early anti-retroviral therapy on the pathogenic changes in mucosal tissues of SIV infected rhesus macaques

**DOI:** 10.1186/1743-422X-9-269

**Published:** 2012-11-14

**Authors:** Jessica Malzahn, Chengli Shen, Lori Caruso, Priyanka Ghosh, Soni Ramachandra Sankapal, Simon Barratt-Boyes, Phalguni Gupta, Yue Chen

**Affiliations:** 1 Department of Infectious Diseases and Microbiology, Graduate School of Public Health, University of Pittsburgh, 130 DeSoto Street, Pittsburgh, PA, 15261, USA; 2 Center for Vaccine Research, University of Pittsburgh, Pittsburgh, Pennsylvania, 15261, USA

**Keywords:** SIV, Gastrointestinal tissue, Immune activation, Viral load

## Abstract

**Background:**

The gastrointestinal tissue plays an important role in the pathogenesis of HIV/SIV infection and serves as a viral reservoir in infected individuals under antiretroviral therapy (ART). However, the effect of ART administration in the very early stage of infection on HIV/SIV replication and pathogenesis in gastrointestinal tissue has not been fully studied. In this current study, rhesus monkeys infected with SIV were treated with ART starting at day 7 post-infection. The effect of early ART on SIV replication and infection-related pathogenic changes in mucosal tissues of the infected monkeys was examined.

**Methods:**

Nuclear acids were extracted from snap frozen ileum and colon tissues and mesentery lymph nodes from SIV infected monkeys with or without ART. SIV RNA and DNA loads as well as levels of CD3, CD4 and cytokine mRNA were measured by PCR and RT PCR from the isolated nuclear acids. Tissue sections were stained by immuno-fluorescence labeled antibodies for CD3 and CD4.

**Results:**

Without ART treatment, these monkeys underwent a mild SIV infection with low viral loads and slightly decreased CD4^+^ T cell counts in peripheral blood. In ART treated monkeys, SIV RNA loads were undetectable in blood with normal CD4^+^ T cell counts, however, SIV RNA and DNA were detected in the intestinal tissues and mesentery lymph nodes although the levels were lower than those in untreated monkeys. The levels of CD3 and CD4 positive cells in the tissues were similar between the infected untreated monkeys and infected ART treated monkeys based on RT-PCR and immune-fluorescence staining of the tissue sections. Furthermore, compatible levels of IL-6, TNF-a, IL-1b and MyD88 mRNAs were detected in most of intestinal tissues and mesentery lymph nodes of infected ART treated and infected untreated monkeys.

**Conclusions:**

These results suggest that early ART administration could not effectively inhibit SIV replication in intestinal tissues and mesentery lymph nodes and could not reduce the immune activation induced by SIV infection in the intestinal tissues.

## Background

The gastrointestinal (GI) tract harbors a majority of lymphocytes both in human and non-human primates. Approximate 40-60% of T lymphocytes in the GI tract are CCR5^+^ CD4^+^ T cells, the main target cells for HIV/SIV infection and replication [1-4]. The gut associated lymphoid tissue (GALT) plays an important role in the pathogenesis of HIV infection and AIDS development. During the early and chronic phases of HIV/SIV infection, HIV/SIV preferentially replicates in the GALT, leading to CD4^+^ T cell depletion, especially Th17 CD4^+^ T cells, local immune activation and mucosal barrier dysfunction [5,6]. The pathogenic changes in GI tissue result in microbial and microbial-product translocation and systemic immune activation, which propels disease progression. In long-term non-progressors (LTNP) and antiretroviral therapy (ART) treated patients, the GALT serves as a viral reservoir which poses a great obstacle in virus eradication from HIV infected individuals [1,7,8].

In the early stage of HIV/SIV infection, regardless of the route of infection, the virus infects gastrointestinal tissues and quickly establishes a viral reservoir. When HIV infected patients are treated with ART, viral loads in peripheral blood decline quickly to an undetectable level. However, viral DNA and RNA can still be detected in lymphoid and gastrointestinal tissues, indicating that virus actively replicates in these tissues in spite of the ART treatment [9,10]. However, in most of these studies, infected patients were treated with ART in their chronic phase of infection.

It is still unclear why ART is unable to effectively control and eradicate HIV from the gastrointestinal tissues. It is speculated that ART administration at a very early stage of infection could more effectively control HIV replication in GI compartments in which HIV is just establishing productive infection. Unfortunately, limited studies have been reported about the effect of early ART treatment on HIV infection and pathogenesis in GI tissues. This information is very important for clinicians to design an effective therapeutic strategy. Macaques infected with SIV provide good animal models for studying HIV with different treatment strategies since it is possible to examine host and viral responses to early ART treatment in different tissue compartments at different times post infection.

In this study, GI tissues from SIV infected rhesus monkeys with or without ART administration at very early phase of infection were studied to explore the effectiveness of early ART administration on viral loads and other pathogenic changes in gastrointestinal tissues.

## Results

### SIV RNA/DNA loads in blood and GI tissues from infected monkeys with or without ART

**Figure 1:**
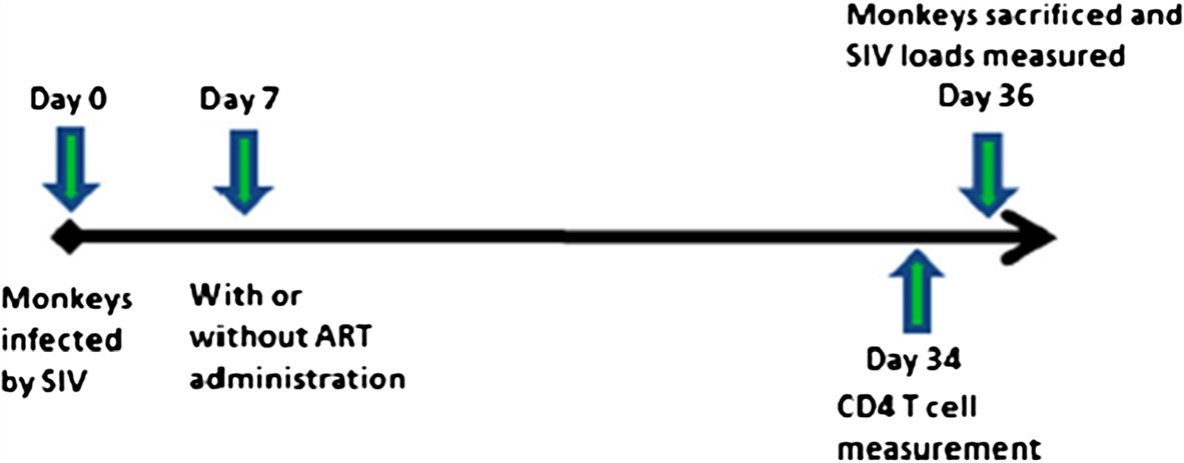
Experimental Schedule of SIV infection and ART Administration.

**Figure 2:**
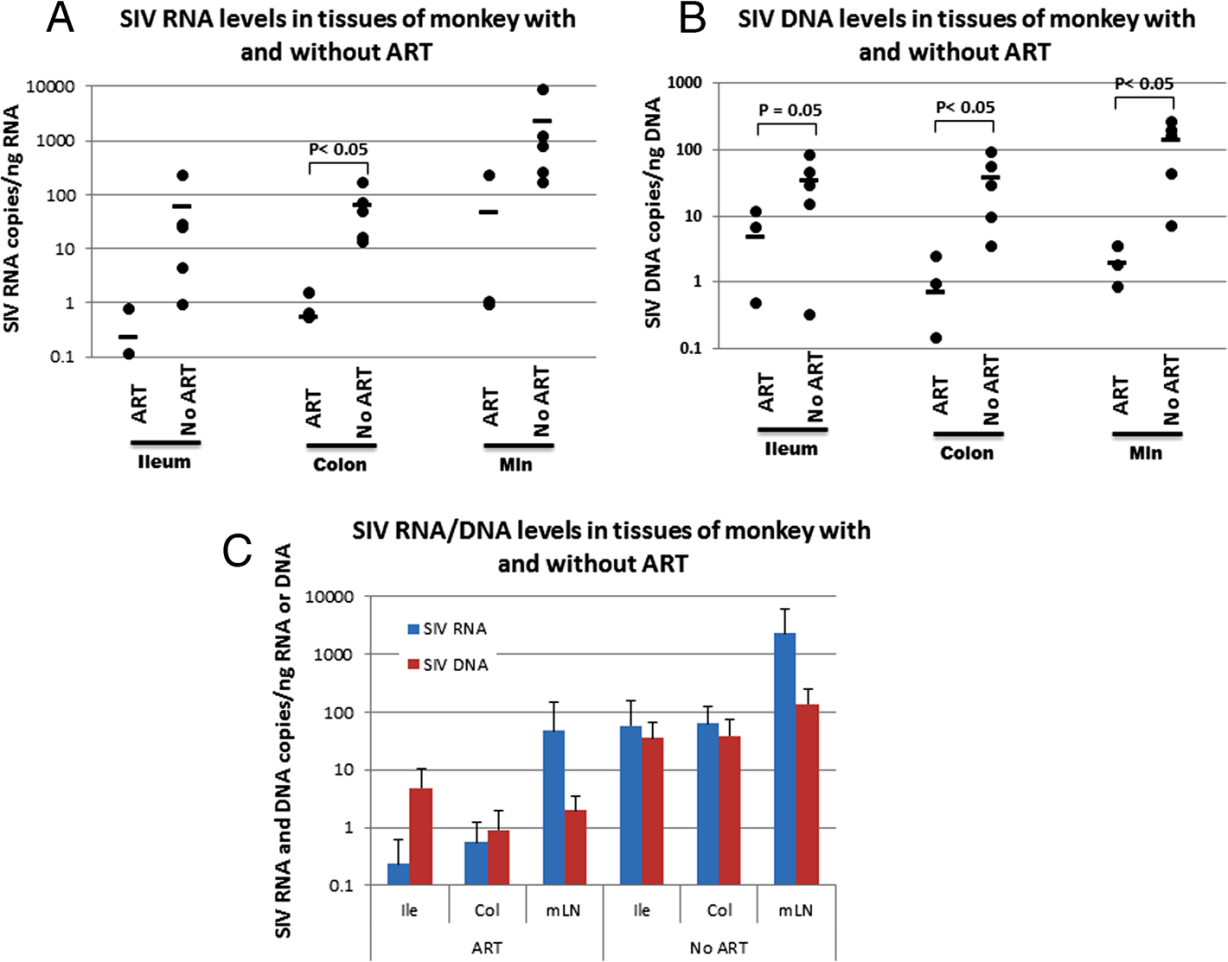
**SIV RNA (A) and DNA (B) loads in ileums, colons and mesentery lymph nodes from SIV infected ART monkeys and SIV infected untreated monkeys.** Nuclear acids were extracted from the tissues and amplified by real time PCR and RT-PCR with SIV specific primers and probe. (**C**) Comparison of viral RNA and DNA levels detected in the same tissue.

Ten rhesus macaques were infected by intravenous inoculation with 100 TCID_50_ of the pathogenic isolate SIVm251. Starting at day 7 post infection, five monkeys were treated with ART--two reverse transcriptase inhibitors (PMPA and FTC, see Materials and methods section for details) and five monkeys were untreated as controls. All monkeys were sacrificed on day 35 post infection (Figure 1). Plasma viral loads and CD4^+^ T cell counts of all the experimental monkeys around sacrificing time are listed in Table 1. The SIV RNA loads in the blood of SIV infected monkeys without ART range from 4.2 × 10^3^ to 5.2 × 10^4^/ml, which indicates these monkeys had a mild SIV infection based on previous reports [11,12]. The mild infection was also evidenced by the moderate CD4^+^ T cell decline in peripheral blood (average CD4^+^ T cells 441/mm^3^). No viral RNA was detected in blood of the infected monkeys with ART, indicating ART effectively inhibited SIV replication. Since gastrointestinal tissues have been implicated to be the major viral reservoir, we evaluated the effect of early ART intervention on viral loads of gastrointestinal tissue in the SIV infected monkeys with or without ART. The SIV RNA and DNA loads in two intestinal locations (ileum and colon) and mesentery lymph nodes (mln) were measured after 35 days of SIV inoculation. As expected, both SIV RNA and DNA were detected in mln, ileums and colons from infected, ART naïve monkeys with detectable plasma viral RNA loads. However, in the five SIV infected monkeys with ART, both SIV DNA and RNA were also detected in mln, ileums and colons despite the undetectable plasma viral loads (Figure 2A and B). Although SIV RNA/DNA was not always detectable in every gut tissues measured, all ART monkeys were found to harbor SIV DNA/RNA in the gastrointestinal tissues. Compared to those of infected monkeys under therapy, the SIV RNA loads in all tissues of ART naïve monkeys were higher, however, only the viral RNA loads in colons of ART naïve monkeys were statistically significantly higher than those in ART monkeys (Figure 2A). SIV DNA loads in all the tissues of ART naïve monkeys are statistically significantly higher than those in ART treated monkeys (Figure 2B). Interestingly, more SIV DNA was detected than viral RNA in the intestinal tissues from ART monkeys, whereas, approximately equal amounts of viral RNA and DNA were detected from ART naïve monkey tissues (Figure 2C). These results indicate that ART administered on day 7 post infection could not block SIV from establishing the viral reservoir in mln and intestinal tissues. The results also suggested that the antiretroviral drugs might penetrate into and partially block SIV replication in the tissues. However, the drug concentrations in the intestinal tissues might not be high enough to totally block SIV replication and local immune cells might not remove the infected cells in the intestinal tissues as suggested in a recent review by Dr. Cohen [13].

**Table 1:** Infection status and Clinical findings in Rhesus Macaques

**Monkey ID**	**Infection stage and treatment**	**Inoculation strain of SIV**	**Route of Infection**	**Plasma viral loads (copies/ml)**	**CD4 T cells/mm3**
M126-08	Acute +ART	SIVmac251	IV	<200	749
M127-08	Acute +ART	SIVmac251	IV	<200	829
M129-08	Acute +ART	SIVmac251	IV	<200	679
M132-08	Acute +ART	SIVmac251	IV	<200	1682
M134-08	Acute +ART	SIVmac251	IV	<200	332
M128-08	Acute	SIVmac251	IV	7343	273
M130-08	Acute	SIVmac251	IV	52361	422
M133-08	Acute	SIVmac251	IV	4200	714
M139-08	Acute	SIVmac251	IV	6716	427
M140-08	Acute	SIVmac251	IV	21974	367

### Quantitation of CD3 and CD4 positive cells in intestinal tissues with or without ART

**Figure 3:**
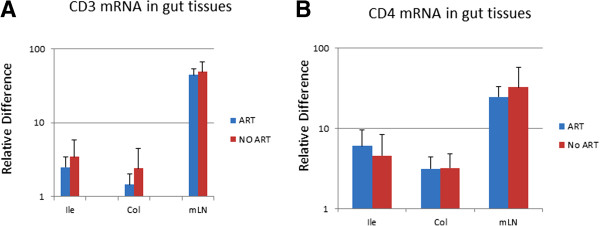
**Levels of CD3 mRNA (A) and CD4 mRNA (B) detected in ileums, colons and mesentery lymph nodes from SIV infected ART monkeys and infected untreated monkeys.** Nucleic acid were extracted from the tissues and amplified by RT real time PCR with CD3 and CD4 specific primers and probes.

**Figure 4:**
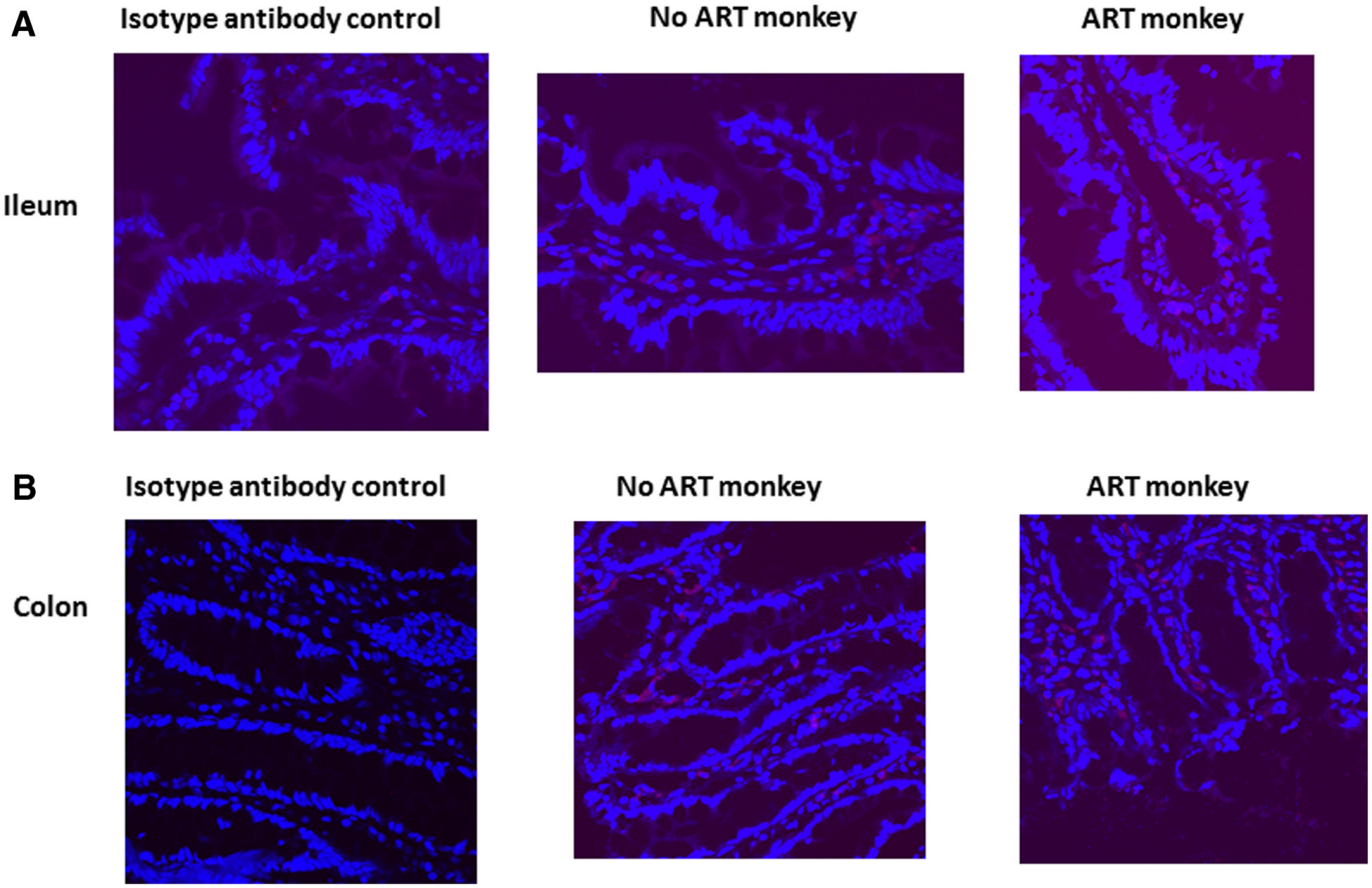
**Distribution of CD4 positive cells in ileums (A) and colons (B) from the SIV infected ART monkey M129-08 and one SIV infected unteared monkey M139-08.** Tissue sections were prepared from snap-frozen tissues and CD4 positive cells were detected by immunufluorescent staining with anti-CD4 antibody followed by confocal miroscopy.

**Figure 5:**
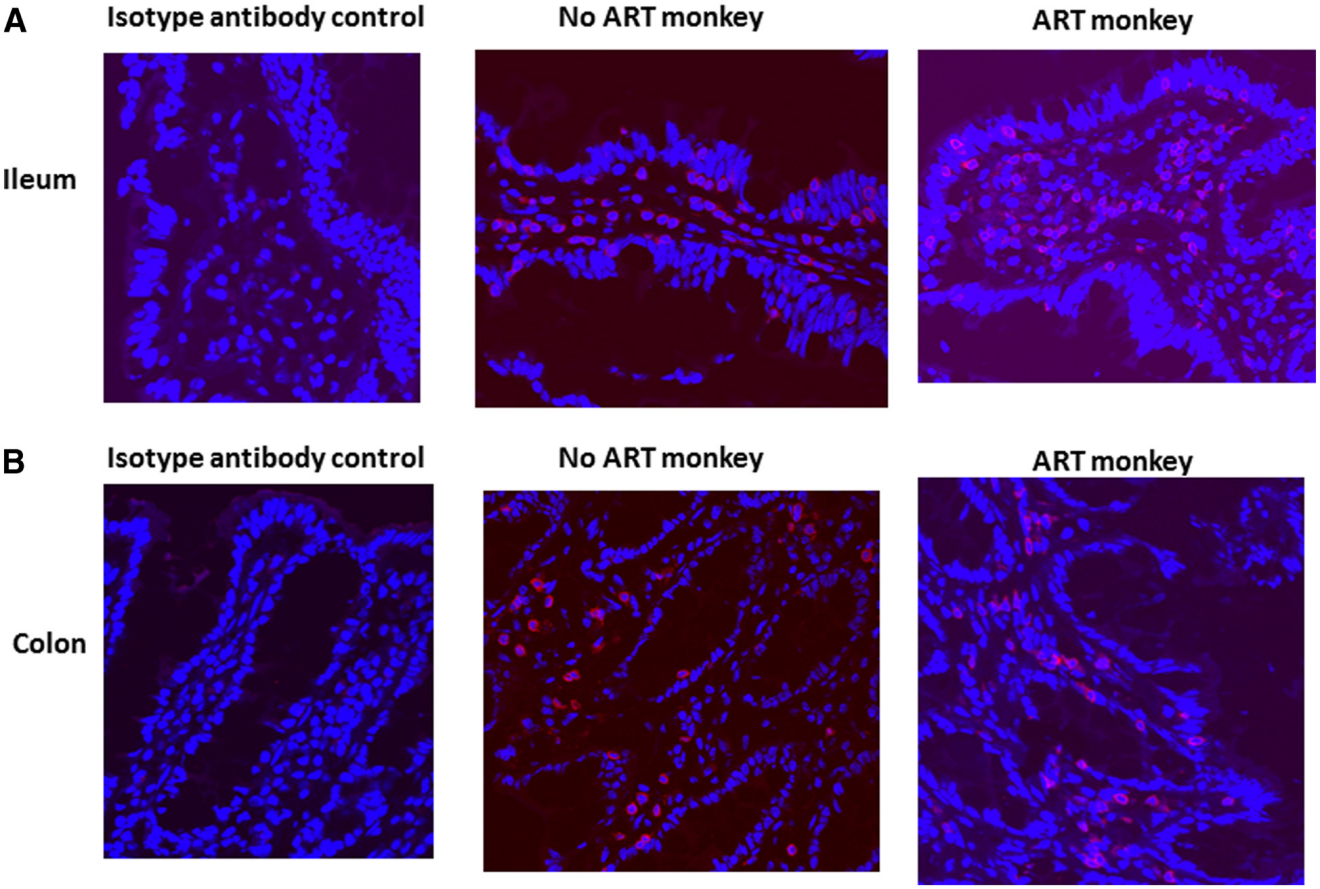
**Distribution of CD3 positive cells in ileums (A) and colons (B) from the SIV infected ART monkey M129-08 and one SIV infected unteared monkey M139-08.** Tissue sections were prepared from snap-frozen tissues and CD3 positive cells were detected by immunufluorescent staining with anti-CD3 antibody followed by confocal miroscopy.

Monitoring CD4 positive cell quantity in the tissues is the most frequently used method for evaluating local HIV/SIV induced pathogenic changes. Since only snap-frozen tissues from these monkeys were available to us, it was very difficult to isolate the immune cells from the tissues and phenotype them by flow cytometry. Therefore as an alternative approach we measured the levels of CD4 and CD3 mRNA in the mln and intestinal tissues by real time RT PCR, which served as a surrogate measurement for expression of CD4 and CD3 in cells of the tested tissues. The results presented in Figure 3 showed that the levels of CD4 and CD3 mRNA in the intestinal tissues from infected, ART treated monkeys were similar to those of the intestinal tissues from infected, ART naïve monkeys. In addition, the quantitation of CD3 and CD4 positive cells in the intestinal tissues were further assessed by immuno-fluorescent staining in the tissue sections from two infected ART treated monkeys (M129-08 and M132-08) and two infected ART naïve monkeys (M139-08 and M140-08). The representative results from monkey M129-08 and M139-08 were included in Figures 4 and 5. No significant differences in the numbers of CD3 and CD4 positive cells in the tissue sections were observed between SIV infected, ART treated monkeys and ART naïve monkeys. These results are not unexpected since early administration of ART did not effectively inhibit viral replication in intestinal tissues.

### Cytokine profiles in SIV infected intestinal tissues with/without ART

**Figure 6:**
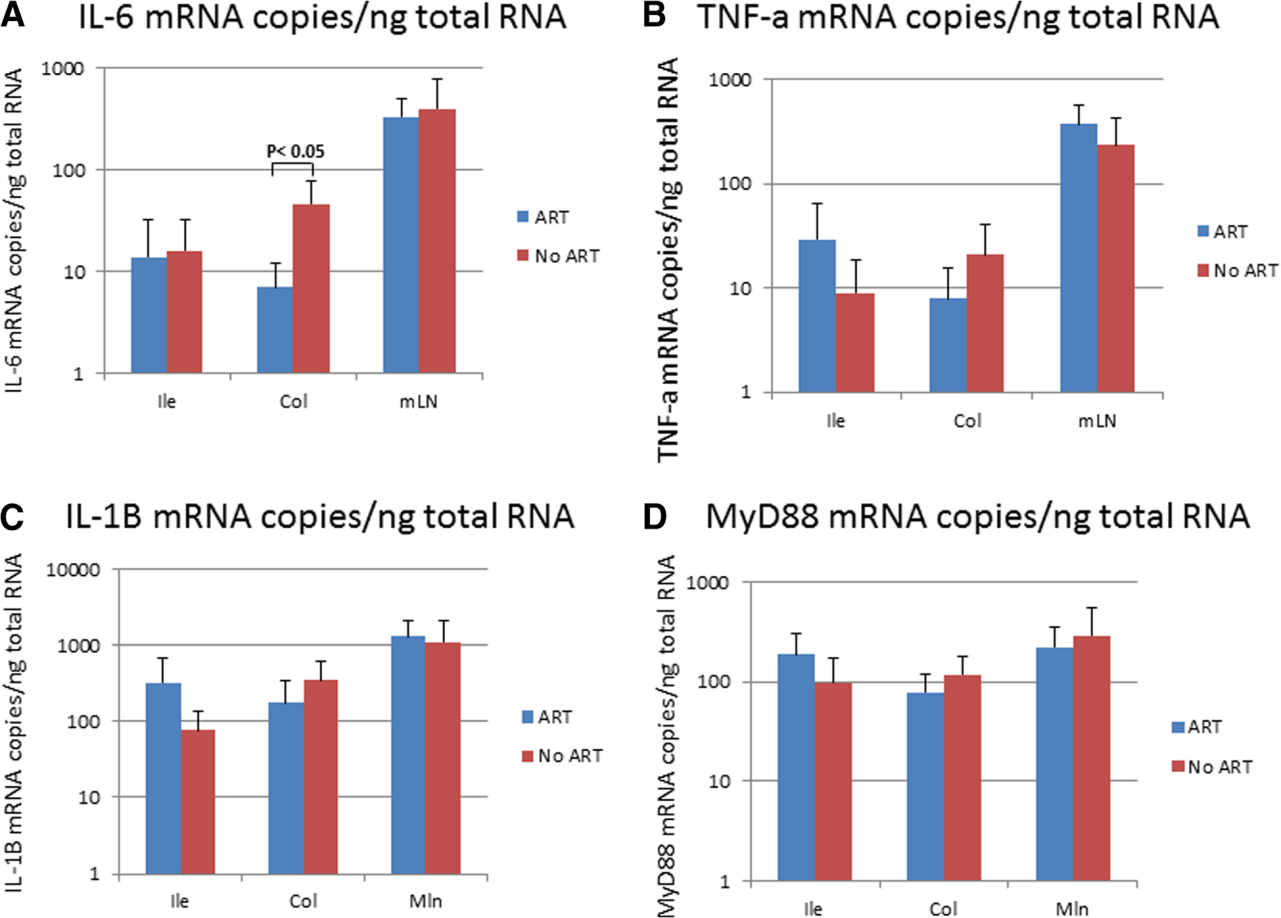
**Levels of IL-6 mRNA (A),TNF-a mRNA (B), IL-1b (C) and MyD88 mRNA (D) detected in ileums, colons and mesentery lymph nodes from SIV infected ART monkeys and infected untreated monkeys.** Nucleic acids were extracted from the tissues and amplified by RT real-time PCR with IL-6, TNF-a, IL-1b and MyD88 specific primers and probes.

Inflammatory responses induced by SIV replication in gastrointestinal tissues lead to recruitment and activation of immune cells into the local area enhancing viral replication and tissue damage. To evaluate the effectiveness of early administration of ART on pathogenic changes in the infected intestinal tissues, mRNA of the pro-inflammatory cytokines, IL-6, IL-1β, and TNFα were measured by real time RT PCR in the RNA isolated from the intestinal tissues and mln. Significantly higher IL-6 mRNA levels were detected in the colons from infected ART naïve monkeys compared to those from infected ATR treated monkeys (Figure 6A). No statistical differences in the levels of TNFα and IL-1β mRNA were observed in intestinal tissues from infected ART treated monkeys compared to infected ART naïve monkeys (Figure 6B and C). MyD88, an adapter protein used by TLRs to activate the NF-KB transcription, is one of the important components of innate immune responses. Therefore, the mRNA levels of MyD88 were also monitored in intestinal tissues and mln in monkeys with and without ART. No statistically significant differences in the mRNA levels of MyD88 were detected in the intestinal tissues and mln in ART treated vs ART naïve monkeys (Figure 6D). These results suggest that early ART administration did not efficiently influence GI inflammatory responses.

## Discussion

In HIV/SIV infection, pathogenic changes in the GI tract have been implicated as one of the main causes of disease progression and AIDS development. ART treatment of chronically infected patients decreases plasma viral loads to undetectable levels and maintains those levels for many years. However, in such treated patients, HIV remains replication active in gastrointestinal tissues and lymphoid nodes, and as a result it is very difficult to eradicate HIV from infected individuals. It has been speculated that the concentration of ART in gastrointestinal tissue is much lower than that in blood because the gastrointestinal tissue composition and structures lead to poor drug penetration. Such low levels of ART in tissues could not effectively suppress HIV replication in the tissues, making the gastrointestinal tissue a viral reservoir [13]. Recent studies show that ART treatment of HIV-1 infected persons has a very dramatic impact on reducing HIV-1 transmission rates to their partners [14]. Studies in the macaque models have also demonstrated that early drug treatment delayed or reduced the peak of acute viremia, enhanced antiviral immune responses, and delayed disease progression [15]. Therefore, ART has been suggested to be administered at the early stage of HIV infection to inhibit HIV replication and transmission and prevent pathogenic change in gastrointestinal tract [16,17]. However, due to the hard-to-reach anatomical location, few studies have been performed to monitor the effectiveness of early ART administration on blocking HIV replication and associated pathogenic changes in the gastrointestinal tract, a key area for eradicating HIV infection.

In this current study, five SIV infected monkeys were treated with ART from day 7 post infection to day 35 of infection. Our results showed that SIV replicated in intestinal tissues and mln in spite of the early ART administration. Furthermore, SIV invaded intestinal tissues and established viral reservoirs rapidly and administration of antiviral drugs as early as day 7 post infection could neither efficiently suppress viral replication, nor eliminate the infected cells from this established reservoir. SIV infection in gastrointestinal lymphoid tissues leads to rapid depletion of CD4^+^ cells in the tissues. In the current study, comparable CD4 and CD3 positive cells in the intestinal tissues measured by immuno-fluorescence staining and their mRNA levels in the ART treated and untreated monkeys suggested that early ART administration failed to prevent CD4^+^ cell depletion in gastrointestinal tissues. However, it is also possible that all infected monkeys in this experiment underwent a moderate SIV infection and might experience a slight decline of CD4^+^ cells in intestinal tissues regardless of ART administration. Therefore, it is not expected to observe a significant CD4^+^ T cell depletion in the gut tissues with such a mild and short duration of SIV infection in these experimental monkeys (Dr. Apetrei, University of Pittsburgh, personal communications).

HIV/SIV replication in gastrointestinal tissues induces immune activation and upregulation of multiple cytokines and chemokines, which stimulate inflammatory response and cell death [18-20]. ART administration reduces these pathogenic damages in gastrointestinal tissues of HIV infected patients and SIV infected monkeys [21,22]. One important aspect of immune activation is T cell activation with upregulation of MHC class II and CD38 on cell membranes. Since only snap-frozen intestinal tissues from the experimental monkeys were available for this study, immune cells in the tissue were not able to be evaluated by flow cytometry for immune activation status. However, mRNA levels of inflammatory cytokines were measured in the tissues. Except IL-6 mRNA levels in the colons, there were no differences in mRNA levels of cytokines and MyD88 in intestinal tissues and mln from ART treated monkeys versus ART naïve monkeys. The possibilities for the observed discrepancies are: 1) Severe immune activation might not occur in the tested tissues due to the mild infection; 2) ART could not prevent SIV induced cytokine production; 3) there might be a combination of 1) and 2). Due to the tissue availability, the cytokine and chemokine levels in the tissues were only measured at mRNA level in this study. In a subsequent study, we will measure the cytokine and chemokine levels at protein level in the tissues as well.

HIV/SIV infection with different levels of viral loads and different rates of CD4^+^ T cell decline is observed in an infected population. Most studies have focused on the severe cases of HIV/SIV infection. In this study, we reported mild cases of SIV infection and provided a snapshot of the SIV induced pathogenic changes in intestinal tissues with and without early ART administration, which is less studied, but very important since many people have contracted mild HIV infection with low viral loads and slow CD4^+^ T cell decline.

There are certain limitations in this study by using the frozen tissues from the experimental monkeys: such as, the immune cells could not be isolated from the frozen tissues for precise numeration of CD4 and CD8 T cells in the tissues by flow cytometer analysis, or numeration of SIV infected CD4 T cells, or accurately measuring SIV RNA/DNA copies/number of CD4 T cells. Hence, future studies with fresh intestinal tissues are warranted. Furthermore, due to the limitation of available monkeys, there were no uninfected control monkeys in this study. Future studies that characterize transcriptional profiles of infected with or without ART and uninfected intestinal tissues will be important to the identification of molecular mechanism of pathogenesis and potential targets for therapeutic intervention.

## Conclusions

In this study, the effect of early ART treatment on SIV replication and pathogenic changes in intestinal tissues was evaluated in mild SIV infected monkeys. The results suggest that early ART administration could not effectively inhibit SIV replication in intestinal tissues and mesentery lymph nodes and could not reduce the immune activation induced by SIV infection in the intestinal tissues.

## Materials and methods

### Monkeys, virus infection and sample collection

This study was carried out in strict accordance with the recommendations in the Guide for the Care and Use of Laboratory Animals of the National Institutes of Health. The protocol was approved by the Institutional Animal Care and Use Committee at the University of Pittsburgh (Assurance Number A3187-01). Surgeries were performed under anesthesia induced and maintained with keztamine hydrochloride and medetomidine hydrochloride, and all efforts were made to minimize suffering.

The ten Indian-origin rhesus macaques were infected by intravenous inoculation with 100 TCID_50_ of the pathogenic isolate SIVm251 that had been grown in CEMx174 cells. At day 7 post infection, five monkeys were treated with the Antiretroviral drugs (ART) and five monkeys were untreated as controls. The ART consisted of two reverse transcriptase inhibitors, 9-[2-(phosphonyl-methoxy)propyl] adenine (PMPA) and 29-deoxy-5-fluoro-39-thia-cytidine (FTC). PMPA was administered at 30mg/kg daily from day 7 to day 20 post infection and 20mg/kg daily from day 21 to day 35 by subcutaneous injection; FTC was administered at 30mg/kg daily from day 7 to day 35 post infection via subcutaneous injection. All monkeys were sacrificed on day 35 post infection. CD4^+^ T cell counts and viral loads were measured in peripheral blood two days before and on the sacrificing day respectively (Figure 1). Mesentery lymph nodes, ileum and colon tissues from these monkeys were snap frozen immediately after sacrifice and used in this current study.

### Nucleic acid isolation and RT real time PCR

Two hundred milligrams of each GI tissue was homogenized using the hard tissue disposable probes and TH-01 Homogenizer (OMNI International). The RNA and DNA were isolated simultaneously from the same homogenized tissue mixture using the AllPrep DNA/RNA mini ® kit (Qiagen) according to the manufacturer’s protocol. Twenty nanograms of DNA were used for measuring SIV DNA loads in the tissues. Two hundred nanograms of RNA from the tissues were reversely transcribed with random hexamers by SuperScript II Reverse Transcriptase (Invitrogen) following the protocol provided by the manufacturer. Twenty nanograms of cDNA equivalent were used to quantitate SIV RNA loads and the relative mRNA levels of CD4, CD3 and the inflammatory cytokines by real time PCR. A 25 μl TaqMan® PCR was performed by mixing 20ng of the DNA or cDNA with TaqMan® Universal Mastermix (Applied Biosystems), target specific and 18S primers and probes. The PCR amplification and product detection were performed with a ViiA 7 real time PCR machine (Applied Biosystems). The PCR cycling program consisted of 50 two-step cycles of 15s at 95°C and 60s at 60°C. The nucleic acid from each specimen was amplified in duplicate. A no-template and reverse transcriptase negative controls were included in each PCR run. To accurately quantify the target molecules, endogenous gene 18S was co-amplified using commercially available primers and probe (Applied Biosystems) in each run as a multiplex assay. In the result analysis, the Ct value of a specific target was adjusted by the co-amplified 18S Ct value to compensate the subtle differences in DNA and cDNA inputs between samples. For quantitation of SIV loads in gastrointestinal tissue samples, the serial dilutions of SIV 17E virus stock with known copy number were included in each PCR run to generate a standard curve. SIV loads in the samples were quantitated using the following primers and probe [23], which targeted SIV long terminal repeat (LTR): LTR forward primer - 5’ TGG GAG GTT CTC TCC AGC AC, LTR reverse primer-5’ AAT GGC AGC TTT ATT GAA GAG G and LTR probe- 6FAM 5’ TTC CCT GCT AGA CTC TCA CCA GCA CTT GG TAMRA. To compare the mRNA levels detected in the tissues, a series of three or four times ten-fold dilutions of the samples containing high mRNA levels of CD4, CD3 or inflammatory cytokines were included in each PCR run to generate an arbitrary standard curve for relative quantification of the CD4, CD3 or cytokines in tissue samples. The cytokine primers and probes used for relative quantification were purchased from ABI: CD3—RH02826503; CD4—Rh02621720; IL-6— Rh02789322_m1; TNF-a— Rh02789783_m1; IL-1B— Rh02789775_m1 and MyD88— Rh01573837_m1.

### Immunofluorescence staining and quantitative image analysis

The immunofluorescence staining was performed by incubating tissue sections with either mouse anti-human CD4 (Leica Microsystem) Abs at a 1:20 dilution or Rabbit anti-human CD3 (Dako Cytomation) Abs at a 1:50 dilution, subsequently incubated with Cy3-conjugated secondary Abs respectively. Following nuclear staining with Hoechst (Sigma-Aldrich), sections were mounted with Vectashield mounting medium (Vector Laboratories). Laser scanning confocal microscopy was performed with an Olympus (Tokyo, Japan) FluoView 1000 laser confocal microscope.

### Statistical analysis

Student’s T-Test was used to compare the means of SIV loads and relative mRNA quantity of inflammatory cytokines in the GI tissues from monkeys with or without ART. A two-sided alpha value of 0.05 was set for statistical significance.

## Competing interests

The authors declare that they have no competing interests.

## Authors’ contributions

JM performed the molecular work and participated in drafting the manuscript. CS participated in the study design and molecular work. LC contributed in tissue sample processing, nuclear acid isolation and editing the manuscript. PGhosh participated in molecular work. SS participated in tissue section imaging study. SB participated in study design and manuscript editing. PGupta participated in study design and manuscript editing. YC contributed to the study design, immunostaining of tissue sections and writing the manuscript. All of the authors read and approved the final manuscript.

## References

[B1] Brenchley JM Douek DC HIV infection and the gastrointestinal immune system Mucosal Immunol 2008 1 23 30 10.1038/mi.2007.1 19079157 PMC2777614

[B2] Mehandru S Dandekar S Role of the gastrointestinal tract in establishing infection in primates and humans Curr Opin HIV AIDS 2008 3 22 27 10.1097/COH.0b013e3282f331b0 19372940

[B3] Nilsson J Kinloch-de-Loes S Granath A Sonnerborg A Goh LE Andersson J Early immune activation in gut-associated and peripheral lymphoid tissue during acute HIV infection AIDS 2007 21 565 574 10.1097/QAD.0b013e3280117204 17314518

[B4] Picker LJ Immunopathogenesis of acute AIDS virus infection Curr Opin Immunol 2006 18 399 405 10.1016/j.coi.2006.05.001 16753288

[B5] Dandekar S George MD Baumler AJ Th17 cells, HIV and the gut mucosal barrier Curr Opin HIV AIDS 2010 5 173 178 10.1097/COH.0b013e328335eda3 20543596

[B6] Hunt PW Th17, gut, and HIV: therapeutic implications Curr Opin HIV AIDS 2010 5 189 193 10.1097/COH.0b013e32833647d9 20543599 PMC2917631

[B7] Tincati C Biasin M Bandera A Violin M Marchetti G Piacentini L Vago GL Balotta C Moroni M Franzetti F Clerici M Gori A Early initiation of highly active antiretroviral therapy fails to reverse immunovirological abnormalities in gut-associated lymphoid tissue induced by acute HIV infection Antivir Ther 2009 14 321 330 19474466

[B8] van Marle G Church DL Nunweiler KD Cannon K Wainberg MA Gill MJ Higher levels of Zidovudine resistant HIV in the colon compared to blood and other gastrointestinal compartments in HIV infection Retrovirology 2010 7 74 10.1186/1742-4690-7-74 20836880 PMC2949729

[B9] Yukl S Wong JK Blood and guts and HIV: preferential HIV persistence in GI mucosa J Infect Dis 2008 197 640 642 10.1086/527325 18260765

[B10] Yukl SA Gianella S Sinclair E Epling L Li Q Duan L Choi AL Girling V Ho T Li P Fujimoto K Lampiris H Hare CB Pandori M Haase AT Gunthard HF Fischer M Shergill AK McQuaid K Havlir DV Wong JK Differences in HIV burden and immune activation within the gut of HIV-positive patients receiving suppressive antiretroviral therapy J Infect Dis 2010 202 1553 1561 10.1086/656722 20939732 PMC2997806

[B11] Moore AC Bixler SL Lewis MG Verthelyi D Mattapallil JJ Mucosal and peripheral Lin- HLA-DR+ CD11c/123- CD13+ CD14- mononuclear cells are preferentially infected during acute simian immunodeficiency virus infection J Virol 2012 86 1069 1078 10.1128/JVI.06372-11 22090100 PMC3255858

[B12] Ortiz AM Klatt NR Li B Yi Y Tabb B Hao XP Sternberg L Lawson B Carnathan PM Cramer EM Engram JC Little DM Ryzhova E Gonzalez-Scarano F Paiardini M Ansari AA Ratcliffe S Else JG Brenchley JM Collman RG Estes JD Derdeyn CA Silvestri G Depletion of CD4 T cells abrogates post-peak decline of viremia in SIV-infected rhesus macaques J Clin Invest 2011 121 4433 4445 10.1172/JCI46023 22005304 PMC3204830

[B13] Cohen J HIV/AIDS research. Tissue says blood is misleading, confusing HIV cure efforts Science 2011 334 1614 10.1126/science.334.6063.1614 22194536

[B14] Cohen MS Chen YQ McCauley M Gamble T Hosseinipour MC Kumarasamy N Hakim JG Kumwenda J Grinsztejn B Pilotto JH Godbole SV Mehendale S Chariyalertsak S Santos BR Mayer KH Hoffman IF Eshleman SH Piwowar-Manning E Wang L Makhema J Mills LA de Bruyn G Sanne I Eron J Gallant J Havlir D Swindells S Ribaudo H Elharrar V Burns D Taha TE Nielsen-Saines K Celentano D Essex M Fleming TR Prevention of HIV-1 infection with early antiretroviral therapy N Engl J Med 2011 365 493 505 10.1056/NEJMoa1105243 21767103 PMC3200068

[B15] Van Rompay KK Evaluation of antiretrovirals in animal models of HIV infection Antiviral Res 2010 85 159 175 10.1016/j.antiviral.2009.07.008 19622373

[B16] Granich RM Gilks CF Dye C De Cock KM Williams BG Universal voluntary HIV testing with immediate antiretroviral therapy as a strategy for elimination of HIV transmission: a mathematical model Lancet 2009 373 48 57 10.1016/S0140-6736(08)61697-9 19038438

[B17] Novitsky V Wang R Bussmann H Lockman S Baum M Shapiro R Thior I Wester C Wester CW Ogwu A Asmelash A Musonda R Campa A Moyo S van Widenfelt E Mine M Moffat C Mmalane M Makhema J Marlink R Gilbert P Seage GR III DeGruttola V Essex M HIV-1 subtype C-infected individuals maintaining high viral load as potential targets for the "test-and-treat" approach to reduce HIV transmission PLoS One 2010 5 e10148 10.1371/journal.pone.0010148 20405044 PMC2853582

[B18] Ndolo T Rheinhardt J Zaragoza M Smit-McBride Z Dandekar S Alterations in RANTES gene expression and T-cell prevalence in intestinal mucosa during pathogenic or nonpathogenic simian immunodeficiency virus infection Virology 1999 259 110 118 10.1006/viro.1999.9709 10364494

[B19] Olsson J Poles M Spetz AL Elliott J Hultin L Giorgi J Andersson J Anton P Human immunodeficiency virus type 1 infection is associated with significant mucosal inflammation characterized by increased expression of CCR5, CXCR4, and beta-chemokines J Infect Dis 2000 182 1625 1635 10.1086/317625 11069233

[B20] Reinhart TA Fallert BA Pfeifer ME Sanghavi S Capuano S III Rajakumar P Murphey-Corb M Day R Fuller CL Schaefer TM Increased expression of the inflammatory chemokine CXC chemokine ligand 9/monokine induced by interferon-gamma in lymphoid tissues of rhesus macaques during simian immunodeficiency virus infection and acquired immunodeficiency syndrome Blood 2002 99 3119 3128 10.1182/blood.V99.9.3119 11964273

[B21] George MD Reay E Sankaran S Dandekar S Early antiretroviral therapy for simian immunodeficiency virus infection leads to mucosal CD4+ T-cell restoration and enhanced gene expression regulating mucosal repair and regeneration J Virol 2005 79 2709 2719 10.1128/JVI.79.5.2709-2719.2005 15708990 PMC548479

[B22] Guadalupe M Sankaran S George MD Reay E Verhoeven D Shacklett BL Flamm J Wegelin J Prindiville T Dandekar S Viral suppression and immune restoration in the gastrointestinal mucosa of human immunodeficiency virus type 1-infected patients initiating therapy during primary or chronic infection J Virol 2006 80 8236 8247 10.1128/JVI.00120-06 16873279 PMC1563811

[B23] Fuller DH Rajakumar PA Wilson LA Trichel AM Fuller JT Shipley T Wu MS Weis K Rinaldo CR Haynes JR Murphey-Corb M Induction of mucosal protection against primary, heterologous simian immunodeficiency virus by a DNA vaccine J Virol 2002 76 3309 3317 10.1128/JVI.76.7.3309-3317.2002 11884556 PMC136011

